# Automated classification of group B *Streptococcus* into different clonal complexes using MALDI-TOF mass spectrometry

**DOI:** 10.3389/fmolb.2024.1355448

**Published:** 2024-06-27

**Authors:** Lianfen Huang, Kankan Gao, Huamin Zhong, Yongqiang Xie, Bingshao Liang, Wenjing Ji, Haiying Liu

**Affiliations:** ^1^ Department of Laboratory Medicine, Guangzhou Women and Children’s Medical Center, Guangzhou Medical University, Guangzhou, China; ^2^ Department of Pharmacy Administration and Clinical Pharmacy, School of Pharmacy, Xi’an Jiaotong University, Xi’an, China; ^3^ Clinical Laboratory, The Affiliated Brain Hospital of Guangzhou Medical University, Guangzhou, China

**Keywords:** group B *streptococcus*, clone complexes (CCs), matrix-assisted laser desorption ionization time of flight mass spectrometry (MALDI-TOF MS), main spectrum (MSP), classification group B *Streptococcus*, classification clean version group B *Streptococcus*, classification

## Abstract

**Objectives:**

To evaluate the performance of Matrix-Assisted Laser Desorption/Ionization Time-of Flight Mass Spectra (MALDI-TOF MS) for automated classification of GBS (Group B *Streptococcus*) into five major CCs (clonal complexes) during routine GBS identification.

**Methods:**

MALDI-TOF MS of 167 GBS strains belonging to five major CCs (CC10, CC12, CC17, CC19, CC23) were grouped into a reference set (*n* = 67) and a validation set (*n* = 100) for the creation and evaluation with GBS CCs subtyping main spectrum (MSP) and MSP-M using MALDI BioTyper and ClinProTools. GBS CCs subtyping MSPs-M was generated by resetting the discriminative peaks of GBS CCs subtyping MSP according to the informative peaks from the optimal classification model of five major CCs and the contribution of each peak to the model created by ClinProTools.

**Results:**

The PPV for the GBS CCs subtyping MSP-M was greater than the subtyping MSP for CC10 (99.21% vs. 93.65%), but similar for CC12 (79.55% vs. 81.06%), CC17 (93.55% vs. 94.09%), and CC19 (92.59% vs. 95.37%), and lower for CC23 (66.67% vs. 83.33%).

**Conclusion:**

MALDI-TOF MS could be a promising tool for the automated categorization of GBS into 5 CCs by both CCs subtyping MSP and MSP-M, GBS CCs subtyping MSP-M is preferred for the accurate prediction of CCs with highly discriminative peaks.

## Introduction

*Streptococcus agalactiae*, also known as Group B *streptococcus* (GBS), is a gram-positive coccus that commonly colonizes the female lower genital tract and rectum. Since the 1980s, it has been recognized as a prominent bacterial pathogen causing neonatal invasive infections such as sepsis, meningitis, and pneumonia, resulting in acute illnesses, long-term impairments, and even neonatal death (Nanduri et al., 2019). GBS gestational colonization during late pregnancy may pose a great threat to fetal health as the primary risk factor for preterm birth, spontaneous abortion and neonatal GBS infections (Delara et al., 2023). GBS strains of different lineages differ greatly in pathogenicity and virulence, epidemiology and antibiotic resistance phenotypes (Manning et al., 2009; Tazi et al., 2010; Teatero et al., 2017; Wang et al., 2018; Arias et al., 2019; Gori et al., 2020; Zheng et al., 2020; Le Gallou et al., 2023).

Nowadays, GBS strains have been commonly determined by genoserotyping with serotyping through latex agglutination (LA) assay using the specific surface capsular polysaccharide (CPS) antibodies and PCR amplification of the capsular gene for the molecular epidemiological survailence (Imperi et al., 2010). However, the high expense of genoserotyping makes it difficult for routine clinical analysis. Moreover, GBS strains belonging to identical capsular serotype III or Ib have shown significant differential characteristics in phenotype and genotype (Arias et al., 2019; Wu et al., 2019; Zheng et al., 2020). Multi-locus sequence typing (MLST) has been widely used to examine the genetic lineages of GBS since 2003 (Jones et al., 2003). However, the high expense, time-consuming, labor-intensive and complex procedures of MLST also forbid its clinical application in routine epidemiological surveillance.

Matrix-Assisted Laser Desorption/Ionization Time-of Flight Mass Spectrometry (MALDI-TOF MS) has been widely applied for the fast and accurate clinical identification of microbial species in recent years. MALDI-TOF MS could identify different subspecies (Wang et al., 2022; Liu et al., 2023; Oberg et al., 2023), like automated, fast and accurate prediction of methicillin-resistant *Staphylococcus aureus* (MRSA) clonal complexes (CCs) (Camoez et al., 2016) and GBS subspecies-level typing based on the mass variation of ribosomal subunit proteins (rsp profile) (Rothen et al., 2019). The previous reported GBS subtyping strategies based on MALDI-TOF MS all require manual assessment of the acquired spectra and highly trained personnel with professional tools like ClinProTool software or online GBS serotyper, including peak biomarkers of different GBS sequence types (STs) and serotypes (Lartigue et al., 2011; Lanotte et al., 2013; Lin et al., 2019), the statistical models generated based on MALDI-TOF MS spectrometry for the rapid classification of major GBS serotypes (Ia, Ib, III, V, VI) (Wang et al., 2019) and STs (ST10, ST12, ST17, ST19) (Huang et al., 2020), or the facile machine learning GBS CCs multi-classification model generated through XGBoost algorithm based on the antibiotic susceptibility, serotypes and virulence genes of GBS strains (Liu et al., 2022), making them not suitable for routine clinical application. Unfortunately, an automated and fast method to detect different GBS CCs lineages simultaneous with MALDI-TOF MS microbial species identification has not yet been evaluated. This study aimed to analyze the potential of MALDITOF MS to discriminate the major neonatal GBS lineages in China using an automated approach based on two Biotyper main spectrum (MSP).

## Materials and methods

### Bacterial strains

This investigation comprised 178 GBS strains isolated from infants under 90 days old in China (Ji et al., 2019). All strains have been identified by MLST and serotyping in our previous research (Supplementary Table S1) (Ji et al., 2019).The clinical information of all strains have been checked to assure that isolates corresponding to identical strains were not involved in this study. ST10 strains were specially categorized as CC10, other CCs were determined based on the STs in GBS typing database (https://pubmlst.org/bigsdb?db=pubmlst_sagalactiae_seqdef&page=downloadProfiles&scheme_id=1).Totally, 167 isolates belonging to five major CCs (CC10, CC12, CC17, CC19, CC23) were separated into two sets for assessment of GBS subtyping MSP: 1) a reference set of 67 isolates: ST10 (*n* = 9) from CC10, ST12 (*n* = 10) from CC12, ST17 (*n* = 26) from CC17, and ST19 (*n* = 14) from CC19, ST23 (*n* = 5), ST55 (*n* = 1), ST223 (*n* = 1), ST163 (*n* = 1) from CC23; (ii) a validation set of 100 isolates: ST10 (*n* = 21) of CC10, ST12 (n = 20), ST8 (*n* = 1), ST268 (*n* = 1) of CC12, ST17 (*n* = 29), ST188 (*n* = 1), ST680 (*n* = 1) of CC17, ST19 (*n* = 16), ST27 (*n* = 2) of CC19, ST23 (*n* = 6), ST249 (n = 1), ST88 (*n* = 1) of CC23. An additional set of 11 isolates comprised sporadic GBS STs including:ST24 of CC452 (*n* = 3), ST938 (*n* = 1), ST156 (*n* = 1) and ST2 of CC1 (*n* = 2), and ST103 (*n* = 1), ST4 (*n* = 1), ST651 (*n* = 2) (Supplementary Table S2). This study for analyses of clinical isolates was approved by the Ethics Committee of Guangzhou Women and Children’s Medical Center (2017021915).The patients/participants provided written informed consent to participate in this study (Ji et al., 2019).

### Sample preparation and MALDI-TOF MS data acquisition

GBS strains were cultured on Columbia sheep blood agar at 37°C for 16–18 h, which were then collected to extract protein using ethanol-formic acid extraction method (Lartigue et al., 2009). MALDI-TOF MS was performed on a MALDI Microflex LT (Bruker Daltonics, Bremen, Germany) instrument under the control of FlexControl software.1 µL protein extraction from each strain was spotted on the MALDI target plate (MSP 96 target steel; Bruker Daltonics) and air-dried at room temperature. Each dried protein spot was covered with 1 µL saturated matrix solution of α-cyano-4-hydroxy-cinnamic acid (Bruker Daltonics) in 50% acetonitrile-2.5% trifluoroacetic acid (Fisher Scientific, U.K) and air-dried for further MS analysis. For each strain, 240 laser shots from 40 separate sample spots were automatically collected at 60 Hz (random walk movement). Protein extracts of the reference and validation sets were spotted on a MALDI target plate in eight and two replicates, which were detected three times to obtain 24 and 6 mass spectra for each strain in the reference and validation sets respectively. By using the main spectrum (MSP) identification standard method (mass range 2000–20000 Da) on the MALDI BioTyper (Version 3.1; Bruker Daltonics), mass spectra were acquired in linear positive mode at a laser frequency of 20 Hz. All raw spectra were aligned to the GBS MSP database using the MALDI Biotyper pattern-matching algorithm.

As for the classification of GBS CCs by GBS CCs subtyping MSP relies mostly on the informative peaks with low intensity or a combination of low peaks, the mass spectra of GBS strains with a logarithm score [*log(S)*]≥2.3 during GBS species identification were recommended and preferred to be enrolled into the CCs reference set for better CCs subtyping subclassification, but for validation, mass spectra for certain GBS strains with multiple measurements and scores 2.0–2.3 could also be used for CCs prediction, especially for CC10 and CC17 with specific informative peaks. Specifically, for mass spectra of CC12, CC19, and CC23 strains without good informative peaks, mass spectra with high identification log (scores) ≥2.3 were preferred for more reliable prediction of the subtyping CCs. Detailed pipeline applied to the raw spectra was shown in Supplementary Document S1.

### MALDI-TOF MS data analysis

Mass spectra from the reference set were grouped into 5 CCs classes (CC10, CC12, CC17, CC19, CC23). The multiple spectra of each strain from 123 GBS isolates representative of five major CCs (CC10, *n* = 20; CC12, *n* = 20, CC17, *n* = 49; CC19, *n* = 21; CC23, *n* = 13) were loaded into ClinProTools software (version 3.0; Bruker Daltonics) for model generation and peak analysis. To avoid errors in the statistical calculation of mass spectra through multiple measurements, spectra grouping and similarity selection were enabled before loading spectra. Spectra processing on ClinProTools includes peak selection and calculation of average peak list. Mass to charge ratio values (*m/z*) ranging from 2000 to 10,000 were used. Null spectra exclusion was enabled. The other default settings remained unchanged. The *m/z* values from the average spectrum of major CCs were extracted to identify statistical discriminative peaks when the *p*-value for the Anderson–Darling test was >0.05 and for the t-/ANOVA or Wilcoxon/Kruskal–Wallis test was ≤0.05, or if the *p* values for the Anderson–Darling test and the Wilcoxon/Kruskal–Wallis test were ≤0.05 (Camoez et al., 2016). The average spectrum of each CC class was calculated in order to create pattern recognition models using the Genetic algorithm (GA), the Supervised neural network (SNN), and the Quick Classifier (QC) algorithms (Bruker Daltonics GmbH, 2011). Recalibration was carried out with a 1000 parts per million maximal peak shift and a match to calibrant peaks. Spectra that had not been calibrated were excluded. All peaks in the spectra were picked in model generation. For GA-KNN, GA algorithm was used as a method to select the peak combinations, the maximum number of best peaks was evaluated as 10, 20, and 30 respectively, the maximum number of generations was set to be 500 for GA algorithm to run to assure it wouldn’t not be reached as the stop criteria to halt calculation. The numbers of the k-nearest neighbors (k-NN) evaluated were 1, 3, 5, 7 for each binary class separation. Random mode was chosen for calculating cross validation of generated ML models. The recognition capability and cross validation values were calculated to evaluate the performance of the calculated models. The optimal discriminative peaks and separation weights provided by the best CCs classification model in this study as well as the GBS ST classification models in our previous report (Huang et al., 2020) were taken into consideration for modifying the GBS subtyping MSP to MSPs-M.

**FIGURE 1:**
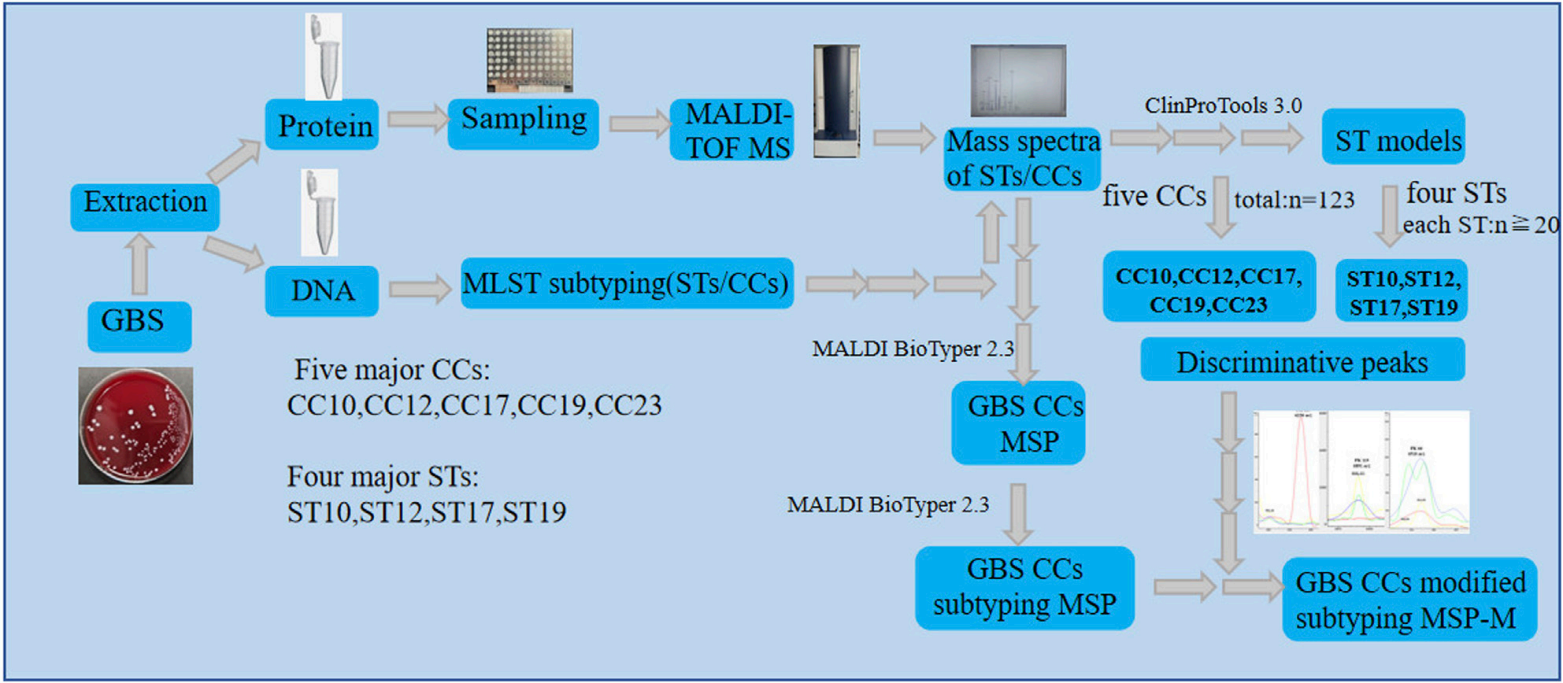
The flow chart of the study design.

MSPs of five major GBS CCs specifies (CC10,CC12,CC17,CC19,CC23) were first created using the BioTyper MSP creation method respectively with default parameters. Then, they were all selected for creating the subtyping MSPs of five GBS CCs specifies using the BioTyper subtyping MSP creation method with default parameters. To generate CCs MSPs-M, the specific weights for peaks in the subtyping MSPs were set to 0 or replaced by the weight values of the informative peaks calculated by optimized algorithms. To assess the performance of CCs subtyping MSP and MSP-M, an external validation using 100 GBS isolates from the validation set was performed. Six different spectra for each strain in the validation set were loaded into the Biotyper software and categorized. The CCs subtyping MSP and MSP-M database were searched for the best match with each mass spectra. A cut-off log score value of 2.4 was recommonded to determine the subtyping prediction of the mass spectra. External calibration of the spectra was performed routinely using Bacterial Test Standard (Bruker Daltonics). A more clear flow chart of the study design was displayed in Figure 1, a more detailed paragraph clarifying both subtyping methods was described in Supplementary Document S1.

## Results

### Creation of GBS CCs classification models

After mass spectra loading on ClinProTools, the automatically selected average mass spectra for each strain of five GBS CCs isolates were used to calculate and generate pattern recognition models by GA, SNN and QC algorithms. Their performances were shown in Table 1. The GA (20)-KNN1 model generated by GA algorithm displayed the highest recognition (100%) and cross-validation rates (80.89%) (Supplementary Table S3). Therefore, it was selected to offer the optimal set of peaks for discriminating GBS 5 CCs. The GA (20)-KNN1 model identified 20 informative peaks (Table 1). Ten out of the twenty differential peaks showed low *p* values (*p* < 0.05) for the Anderson–Darling test, indicating that the data were not normally distributed, hence the *p*-value of the Wilcoxon/Kruskal–Wallis test was preferred above the *p*-value of the t-/ANOVA test to consider them as informative peaks. Another eight peaks showed *p* values for the Anderson–Darling test greater than 0.05 (normally distributed); hence, the t-/ANOVA was favored instead of the Wilcoxon/Kruskal–Wallis test (Table 2). The statistical analysis revealed that the intensity differences of 91 peaks were statistically significant among 5 CCs (Supplementary xls 1).

**TABLE 1:** The 20 discriminative peaks calculated by optimized GA-KNN models.

Peak no	m/z	Start mass	End mass	Weight
85	5,912.52	5,902.01	5,921.5	13.13
20	2,954.93	2,949.46	2,959.65	10.69
34	3,123.77	3,118.31	3,128.26	9.58
99	6,249.64	6,235.49	6,257.76	9.38
107	6,891.86	6,884.04	6,901.19	8.60
80	5,406.58	5,401.79	5,414.21	5.76
108	6,939.44	6,931.65	6,956.7	5.57
126	9,016.36	8,990.26	9,020.59	4.87
84	5,892.30	5,884.00	5,899.85	4.23
115	7,638.17	7,626.24	7,671.42	3.86
109	7,069.67	7,060.48	7,092.88	3.85
97	6,200.27	6,194.04	6,207.35	3.83
127	9,028.71	9,020.59	9,041.13	3.81
151	14,136.74	14,104.33	14,196.14	3.74
141	10,433.64	10,422.95	10,456.57	3.74
153	14,975.44	14,864.58	15,026.86	3.48
143	10,633.69	10,623.58	10,657.52	3.03
128	9,089.34	9,076.91	9,101.10	2.72
74	5,201.43	5,186.68	5,208.33	2.52
23	2,984.67	2,981.11	2,986.23	1.22

**TABLE 2:** Peak statistics for the 20 discriminative peaks calculated by optimized GA-KNN models on CliniProTools 3.0.

no^a^	Mass	DAve	PTTA	PWKW	PAD	CC10	CC12	CC17	CC19	CC23
20	2,954.93	5.54	0.00000218	<0.000001	<0.000001	1.77	1.64	7.18	1.93	5.01
23	2,984.67	3.94	0.186	0.0357	<0.000001	2.84	2.97	3.3	6.51	2.56
34	3,123.77	5.73	0.0000374	0.0000279	<0.000001	6.57	1.03	0.85	0.99	1.47
74	5,201.43	2.7	0.305	0.214	0.492	15.18	16.56	15.08	13.86	13.96
80	5,406.58	4.57	0.0000216	0.0000153	0.0000022	2.03	1.13	2.71	1.9	5.69
84	5,892.3	0.85	0.000678	0.0000672	0.207	1.73	2.08	2.56	1.71	1.95
85	5,912.52	12.42	<0.000001	<0.000001	<0.000001	3.88	4.23	16.3	4.99	11.8
97	6,200.27	1.37	0.0000301	0.0000568	0.82	3.82	4.32	5.1	4.27	3.72
99	6,249.64	14.9	<0.000001	0.0000568	<0.000001	16.24	1.33	1.34	1.41	2.23
107	6,891.86	11.09	<0.000001	0.0000568	0.0000256	7.09	15.75	16.37	18.18	17.68
108	6,939.44	25.31	0.0000871	0.0000279	0.633	48.11	22.8	28.95	40.86	43.18
109	7,069.67	1.15	0.0000231	0.0000659	0.0249	2.53	2.27	2.28	2.93	3.42
115	7,638.17	2.75	0.0223	0.00444	<0.000001	1.4	1.45	1.44	0.98	3.72
126	9,016.36	3.14	0.0000216	0.0000279	0.00105	5.88	6.59	5.31	3.45	4.35
127	9,028.71	1.95	0.0224	0.0164	0.858	6.63	7.02	7.47	8.58	7.21
128	9,089.34	2.42	0.0158	0.0251	0.371	8.89	10.35	8.93	7.93	8.86
141	10,433.64	0.73	0.351	0.241	0.308	3.08	3.65	3.13	3.32	2.91
143	10,633.69	0.28	0.00264	0.00102	0.718	0.81	0.92	0.93	0.74	0.65
151	14,136.74	0.12	0.133	0.111	0.0467	0.35	0.43	0.33	0.33	0.45
153	14,975.44	0.8	0.00489	0.00181	0.714	1.74	2.24	1.99	1.5	1.44

Abbreviations: Mass: m/z value; Weight: relative contribution of each peak to the model; Ave: difference between the maximal and the minimal average peak area/intensity of all classes; PTTA: *p*-value of t-/analysis of variance test; PWKW: *p*-value of Wilcoxon/Kruskal–Wallis test (preferable for non-normally distributed data); PAD: *p*-value of Anderson–Darling test, which gives information about normal distribution (*p*-value AD≤0.05, non-normally distributed; *p*-value AD>0.05, normally distributed); Ave, peak area/intensity average of each class.

^a^ Peak number: correlative numbering of the peak in the average spectra.

**FIGURE 2:**
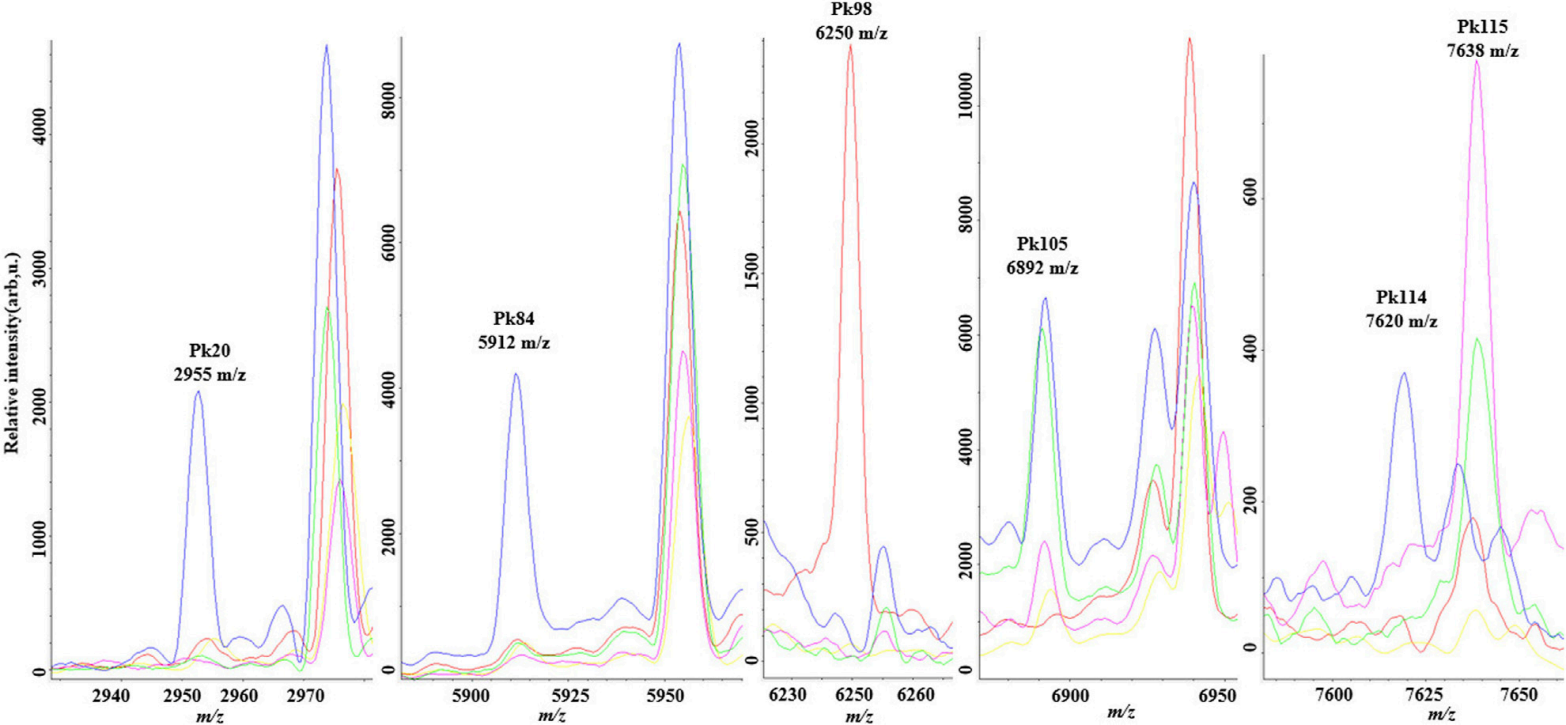
Averaged spectra plots showing the presence or absence of corresponding peak biomarkers for matrix-assisted laser desorption ionization time-of flight mass spectrometry discrimination of the five main Group B streptococcus clonal complexes in the optimal genetic algorithm model. CC10 (red), CC12 (pink), CC17 (green); CC19 (blue), CC23 (yellow). *x*-axis shows the mass per charge ratio values (m/z) and *y*-axis indicates the intensities of peaks expressed in arbitrary intensity units.

**FIGURE 3:**
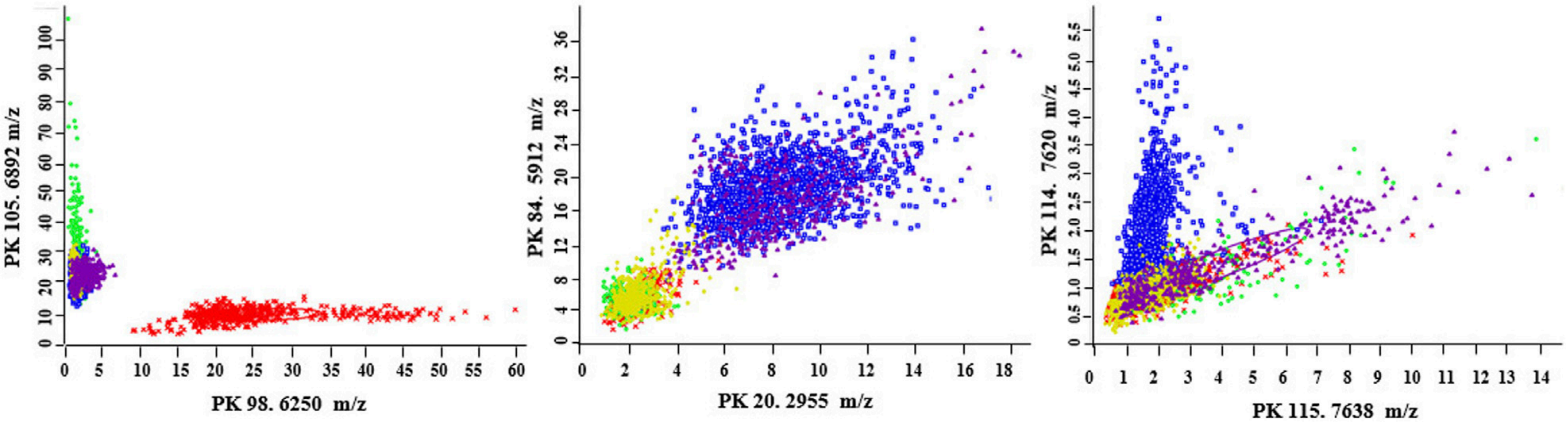
Two-dimensional peak distribution diagrams displaying the pair distribution for the best separating peaks in the optimal genetic algorithm model. The ellipses respresent the standard deviation of the class average of the peak areas/intensities. CC10 (red), CC12 (green), CC17 (blue); CC19 (yellow), CC23 (pink). The peak numbers and m/z values are indicated on the *x*- and *y*-axes, respectively.

The most distinctive peaks for five GBS CCs are shown in Figure 2. In accordance with our previous finding (Huang et al., 2020), a peak biomarker at 6,250 *m/z* was present while a peak at 6,892 *m/z* was absent in all CC10 strains. Two powerful differential peaks at 2,955 *m/z* and 5,912 *m/z* exhibited significantly higher intensity in CC17 strains. A peak biomarker at 7,620 *m/z* of ST17 was proved to be unique for CC17 class, which could coexist with peptide ions 7,635–7,644 *m/z* in some CC17 strains. The peak shift from 7,620 *m/z* to 7,635–7,644 *m/z* was confirmed in all non-CC17 strains as previous researches (Lartigue et al., 2011; Huang et al., 2020). In the paired two-dimensional peak distribution diagrams, the distribution of the two most divergent peaks supported their capacity to differentiate isolates belonging to five major CCs (Figure 3). Pair peaks at 6,250 and 6,892 *m/z* could clearly distinguish mass spectra of CC10 strains, whereas peaks at 2,955 and 5,912 *m/z* could discriminate strains from CC17 and CC23, and peaks at 7,620 and 7,638 *m/z* could separate CC17 strains from other four major CCs (Figure 3).

### Automated classification of GBS spectra into 5 CCs by MALDI biotyper subtyping MSPs and its modified bank MSP-M

24 mass spectra of each strain from the 5 CCs in the reference set were selected to establish the CCs’ specific MSP signature and the associated CCs subtyping MSPs. For GBS CCs subtyping MSPs, the weights of distinguishing peaks among five major CC subclones were automatically set to 0 or the values listed in the supplemental CCs Btmsp files of GBS 5 CCs strains. Meanwhile, referring to the previous study (Camoez et al., 2016), these peaks were also manually edited to generate the CCs subtyping MSPs-M based on the informative peaks in independent GBS STs GA (Huang et al., 2020) and CCs models (Table 1 and Supplementary xls 1). The positive predict values (PPV) of 5 CCs by CCs subtyping MSPs and MSPs-M were listed in Table 3. The PPV for the GBS CCs subtyping MSP-M was greater than the PPV for the subtyping MSP for CC10 (99.21% and 93.65%, respectively), but similar for CC12 (79.55% and 81.06%), CC17 (93.55% and 94.09%), and CC19 (92.59% and 95.37%), and lower for CC23 (66.67% and 83.33%). The peak data of the modified CCs library MSP-M also supported distinctive peaks among five GBS CCs, including peaks at 6,891 (6,888–6,895) *m/z*, 6,250 *m/z*, 3,124 *m/z*, 5,912 *m/z*, 7,620 *m/z* (Supplementary xls 1). Due to lack of statistically informative peaks for CC23 strains, a small specific peak at 6,261 m/z identified in GBS CCs subtyping MSPs for CC12 and CC23, should be kept during peak edition for the generation of GBS CCs subtyping MSP-M for better CCs prediction performance.

**TABLE 3:** Comparison of the validation performance of GBS spectra classification into five major CCs by Biotyper subtyping MSPs and MSP-M automatically.

CC subtyping algolism	CC	No*. of isolates	No. of spectra	Spectra classification					PPV(%)
				CC10	CC12	CC17	CC19	CC23	
subtyping MSPs	10	21	126	118	6	0	0	2	93.65% (118/126)
	12	22	132	2	107	0	15	8	81.06% (107/132)
	17	31	186	0	8	171	6	1	92.19% (171/186)
	19	18	108	0	4	0	103	1	95.37% (103/108)
	23	8	48	0	3	0	5	40	83.33% (40/48)
subtyping MSPs-M	10	21	126	125	1	0	0	0	99.21% (125/126)
	12	22	132	0	105	1	17	9	79.55% (105/132)
	17	32	192	0	7	176	7	2	91.67% (176/192)
	19	18	108	0	8	0	100	0	92.59% (100/108)
	23	8	48	0	6	4	7	31	66.67% (31/48)

## Discussion

Previous study used MALDI-TOF MS to automatically classify four main MRSA CCs lineages (CC5, CC8, CC22, and CC398) based on a Biotyper main spectra (MSP) database with modified CCs informative peaks (Camoez et al., 2016). MALDI-TOF MS was also reported for discriminating four major neonatal GBS STs in China using ClinProTools (Huang et al., 2020). No automated MALDI-TOF/MS based statistical classification methodology has been reported for the fast subtyping of GBS strains yet. In this study, we have evaluated the performance of two subtyping MSP for automated classification of GBS five major CCs (CC10, CC12, CC17, CC19, CC23) in China using MALDI Biotyper alone or in combination with ClinProTools software.

Both GBS CCs subtyping MSP and its modified library MSP-M could be applied for the fast automated prediction of GBS CCs lineages, with clinical acceptable PPV values higher than 90% for CC10, CC17,CC19, and lower PPV of roughly 80% for CC12 and CC23, which would be more clinically acceptable than the traditional non-automated classification strategies described before for GBS subspecies discrimination (Huang et al., 2020).The generation of CCs subtyping MSP is less complicated than its modified library MSP-M, without excessive necessity for obtaining statistically distinguishable peaks and their weights based on classification models with enough strains for each group (n≧20), while these distinguishable peaks and their weights were needed for setting modified CCs library MSP-M. Therefore, the number of strains per CC in the reference set could be as few as five during the generation of CCs MSP, facilitating the possible recognition of certain CCs like CC23 without enough strains (n≦20). The insufficient number of neonatal GBS CC23 strains (5 < n < 20) ultimately resulted in lack of statistically distinguishable peaks specific for ST23 (Huang et al., 2020) and subsequent poorer modification of informative peak parameters of CC23 than other CCs in CCs subtyping MSP-M, leading to a poorer performance of CCs subtyping MSP-M (PPV 66.67%) than MSP on CC23 prediction.

The CCs subtyping MSP is effective at identifying the GBS major CCs automatically, especially for CC12, CC19 and CC23 that lack effective informative peaks. Similar as the previous modified CCs MSP for MRSA that relies on a robust statistical analysis and the automated use of MALDI-TOF/MS to discriminate the major MRSA clonal lineages (Camoez et al., 2016), the modified GBS CCs MSP-M library is an promising way for GBS CCs prediction, facilitating their correct recognition especially for CC10 and CC17 subspecies with biomarker peaks like *m/z* 6,250 (Huang et al., 2020) or *m/z* 7,620 (Lartigue et al., 2011) respectively, with mass spectra of log scores over 2.4. Therefore, although it is more time-consuming for creating the modified CCs MSP-M, the classification performance of CCs MSP-M would be more stable than the unmodified CCs MSP for GBS CCs especially CC10, since identification by CCs MSP-M is less affected by the mass spectra score and the equipment status of MALDI-TOF mass spectrometry (data not shown), allowing better clinical acceptability among different laboratories than the previous GBS subtyping strategies (Lartigue et al., 2011; Huang et al., 2020; Liu et al., 2022).

**FIGURE 4:**
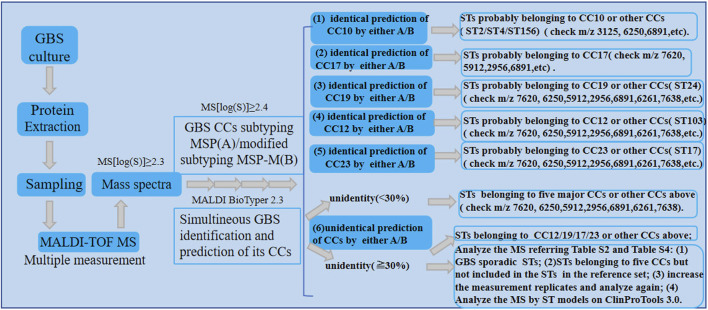
The preliminary procedure for the recognizing of GBS CCs by GBS mass spectra according to the established GBS CCs subtyping MSP and MSP-M models.

However, due to the reduced peak intensity at 5,912 *m/z* or the absence of its unique peak at 7,620 *m/z*, CC17 strains may be mistakenly categorized as non-CC17 strains like CC23 or CC19 by both CCs subtyping MSP and MSP-M. Moreover, the CCs subtyping MSP and MSP-M mistakenly identify isolates from other sporadic CCs with log scores below 2.4 and over 2.4 respectively (Supplementary Table S2). For example, a small number of isolates from CC1 (ST2, *n* = 2; ST156, *n* = 1) and ST4 (*n* = 1) were mistakenly assigned to CC10 with log score values below 2.4. This is probably due to the existence of CC10 peak biomarker *m/z* 6,250 (Huang et al., 2020) in those CCs. Besides, Both methods also mistakenly classified STs belonging to five major CCs which were not included in the STs in reference set with log scores over 2.4 (Supplementary Table S4). To overcome this limitation, representative ST strains belonging to different CCs should be included in the reference set as more as possible to improve the performance of GBS CCs subtyping MSP and MSP-M during the subspecies library creation.According to the CCs subtyping MSP, a log score of mass spectra greater than 2.4 was preferred for more accurate prediction of GBS CCs subtypes. Moreover, multiple measurements of GBS strains were preferred to obtain more mass spectra to be enrolled into the validation set, then the CCs could be categorized according to the major classification results of each GBS strain if possible, which helps to decrease the incorrect prediction rate and improve the performance of GBS CCs predication. To facilitate the application of the established GBS CCs subtyping MSPs and MSP-M models, a preliminary reference procedure for the roughly recognizing of GBS CCs by both methods was displayed in Figure 4.

In summary, we have compared the performance of two GBS CCs subtyping MSPs for the automated GBS CCs categorization, providing suggestions for better optimization of peak parameters and creation of GBS CCs subtyping MSP. This automated MALDI-TOF MS approach could be implemented in routine microbiology labs with MALDI-TOF mass spectrometry simultaneous with microbial identification, allowing a fast identification of GBS CCs. Moreover, CC17 and CC19 were the predominant CCs for neonatal meningitis (Manning et al., 2009; Ji et al., 2019) and pregnant women colonizers (Teatero et al., 2017), which tended to be levofloxacin susceptible but tetracycline resistance or levofloxacin-resistant but tetracycline susceptible (Wang et al., 2018) respectively, CC10 showed high radezolid MICs (Zheng et al., 2020) and FQ resistance (Arias et al., 2019). Therefore, the automated and fast subtyping of GBS CCs will permit a timely initiation of optimal antibiotic selection for the perinatal and neonatal GBS infections, assisting for better GBS prevention and control clinically. Subtyping MSP could be a promising tool for the automated prediction of microbial subtypes in the future.

## Data Availability

The original contributions presented in the study are included in the article/Supplementary Material, further inquiries can be directed to the corresponding author.
