# Supplementary material for: Automated classification of group B Streptococcus into different clonal complexes using MALDI-TOF mass spectrometry
Source: Front Mol Biosci. 2024 Jun 27;11:1355448. doi: 10.3389/fmolb.2024.1355448 (PMC11236597; doi:10.3389/fmolb.2024.1355448)
Supplement: Supplementary file 1 [file DataSheet1.zip › Data Sheet 1/Supplementary Document 1-a pipeline applied to the protein spectra and more detailed paragraph clarifying both subtyping methods.pdf]

1.Supplementary information about the pipeline applied to the protein spectra

Before collection of spectra, it was necessary to check a good status of the MALDI-TOF MS equipment, to determine that whether a professional maintenance of equipment is necessary by engineers. Raw protein spectra of GBS strains from the reference set were collected on a MALDI Microflex LT (Bruker Daltonics, Bremen, Germany) instrument under the control of FlexControl software (version 3.0; Bruker Daltonics). Using the MALDI Biotyper (Version 3.1; Bruker Daltonics) pattern-matching algorithm, all raw spectra were aligned to GBS MSP database. Mass spectra having a logarithm score [*log(S)*] $\geqslant$ 2.3 during GBS species identification were enrolled for statistical peak analysis, model generation, validation, and subtyping MSP to get better CCs subtyping performance in both reproducibility and accuracy. MSP identification standard method included a frequency threshold of 50 for adjusting spectra, a frequency threshold of 5 for score calculation, a maximal mass error of 2000 for raw spectrum, a desired mass tolerance of 250 for the adjusted spectrum, a desired minimum peak frequency of 25%, an accepted mass tolerance of 600 for each peak, and an intensity of 0.25 for the correction function.

According to the special setting of the calculation models described in detail in the ClinProTools user manual (version 3.0; Bruker Daltonics), only one mass spectra were applied for all GBS strains from the reference training set in our previous report for classification model generation and peak analysis. Mass spectrometric multiple measurements (mm) of a protein sample from the same strain were carried out to improve measurement quality. In this study, all multiple measured spectra of each GBS isolate were opened on Flex Analysis software. The abnormal spectra of each GBS isolate that were obvious outlier the major distribution of its all spectra. were deleted. Then, all the remaining spectra were processed via baseline subtraction and spectra smoothing. The multiple measured spectra of each GBS strain in the reference set were grouped by different CCs and loaded into ClinProTools for model generation and peak analysis. To avoid errors in the statistical calculation of mass spectra through multiple measurements, spectra grouping and similarity selection were enabled during

loading spectra. Spectra processing included peak selection and calculation of average peak list were processed automatically. Null spectra exclusion was enabled. The other default settings remained unchanged.

Classification models were generated for performance evaluation through three opened algorithms in ClinProTools, including Genetic Algorithm-K Nearest Neighbor (GA-KNN), Supervised Neural Network (SNN), or QuickClassifier (QC) (Bruker Daltonics GmbH, 2011). All peaks in the spectra were picked in model generation. For GA-KNN, GA algorithm was used as a method to select the peak combinations, the maximum number of best peaks was evaluated as 10, 20 and 30 respectively, the maximum number of generations was set to be 500 for GA algorithm to run to assure it wouldn't not be reached as the stop criteria to halt calculation (Bruker Daltonics GmbH user manual, 2011). The numbers of the k-nearest neighbors (k-NN) evaluated were 1, 3, 5, 7 for each binary class separation (Bruker Daltonics GmbH user manual, 2011). Random mode was chosen for calculating cross validation of generated ML models (Bruker Daltonics GmbH user manual, 2011). The recognition capability and cross validation values were calculated to evaluate the performance of the calculated models. The informative peaks and the corresponding weight produced by the optimized classification models were recorded for further MSP modification. Moreover, the informative peaks and the corresponding weight produced by the optimized classification models for GBS ST10, ST12, ST17 and ST19 in our previous report were recorded for further MSP modification either. One mass spectra of strains ST10 and non-ST10, ST12 and non-ST12, ST17 and non-ST17, ST19 and non-ST19 were selected and pretreated similarly, then they were loaded into ClinProTools 3.0 for generation of GBS ST10, ST12, ST17 and ST19 classification models respectively with similar procedures as measured above.

2.A paragraph clarifying both GBS CCs subtyping MSP and MSPs-M methods

Mass spectra of all GBS strains in the reference set were taken for MSP creation. MSPs representing each GBS CC were created respectively using the BioTyper MSP creation method on MALDI BIOTYPER software, using the BioTyper MSP creation method's default parameters, which included a maximum mass error of 2000 for each

single raw spectrum, a desired mass tolerance of 200 for the MSP, a desired minimum peak frequency of 25%, and a maximal desired peak number of 70 for the MSP. The ion peak list for GBS specie identification includes 70 peaks with 100% weight values but differential frequencies and intensities between 2000 and 20000 Da. For better classification, the mass spectra of different GBS strains were taken to generate different GBS strain MSPs, and the mass spectra of the different GBS strains belonging to the same CC were taken as a whole to generate a CC MSP respectively. Then all created GBS CC-specific and strain MSPs were selected for the creation of GBS CCs subtyping MSPs by resetting the weights of peaks to 0% or certain values automated calculated on the MALDI BIOTYPER software, using the BioTyper subtyping MSP creation method with default parameters, which included a mass range of 3000 to 15000 Da, a maximum number of peaks of 100, a maximum intensity of 1, a maximum intensity influence of 1, a minimum intensity of 0.05, and a minimum intensity influence of 0.5. The weights of peaks in GBS CCs subtyping MSPs could be manually edited to create CC subtyping MSPs-M by resetting their value to 0% or replaced by the weight values of the informative peaks calculated by optimized GA-KNN model (Table 2).

To make the generation of GBS subtyping MSP and MSP-M easier, we found the spectra of all CC10 strains could be selected to set one CC-specific MSPs, as CC10 had a peak biomarker at 6250 *m/z* in all CC10 strains. But for CC17 with a peak biomarker at 7620 *m/z* that only shows in part of CC17 strains, it was recommended that mass spectra of these strains were chosen to set CC17 MSPs respectively. For GBS subclones of CC12, CC19, CC23 which had no obvious peak biomarkers, the spectra of CC12, CC19, CC23 subclones could be selected by strains respectively and CC type totally. As for the obvious worse predication performance of the STs for the major CCs but not included in the reference set (Table S3), the spectra of these rare STs that belong to five major CCs should be chosen respectively to create GBS CC-ST-specific MSPs to increase the predication performance of GBS CCs.
