# Supplementary material for: Automated classification of group B Streptococcus into different clonal complexes using MALDI-TOF mass spectrometry
Source: Front Mol Biosci. 2024 Jun 27;11:1355448. doi: 10.3389/fmolb.2024.1355448 (PMC11236597; doi:10.3389/fmolb.2024.1355448)
Supplement: Supplementary file 1 [file DataSheet1.zip › Data Sheet 1/Supplementary Tables(Table S1-S4).docx]

| **MLST** | **Serotype** | | | | | | | |
| --- | --- | --- | --- | --- | --- | --- | --- | --- |
|  | **Ia** | **Ib** | **III** | **Ⅳ** | **V** | **VI** | **Total** | **CC** |
| 2 |  |  |  |  | 1 | 1 | 2 | **CC1** |
| 4 | 1 |  |  |  |  |  | 1 | **/** |
| 8 |  | 1 |  |  |  |  | 1 | **CC12** |
| 10 |  | 30 |  |  |  |  | 30 | **CC10** |
| 12 |  | 30 |  |  |  |  | 30 | **CC12** |
| 17 | 1 |  | 54 |  |  |  | 55 | **CC17** |
| 19 |  | 1 | 27 |  | 2 |  | 30 | **CC19** |
| 23 | 10 |  | 1 |  |  |  | 11 | **CC23** |
| 24 | 1 |  | 2 |  |  |  | 3 | **CC452** |
| 27 |  |  | 1 |  | 1 |  | 2 | **CC19** |
| 55 | 1 |  |  |  |  |  | 1 | **CC23** |
| 88 | 1 |  |  |  |  |  | 1 | **CC23** |
| 103 | 1 |  |  |  |  |  | 1 | **/** |
| 156 |  | 1 |  |  |  |  | 1 | **CC1** |
| 163 |  |  |  |  | 1 |  | 1 | **CC23** |
| 188 |  |  | 1 |  |  |  | 1 | **CC17** |
| 223 | 1 |  |  |  |  |  | 1 | **CC23** |
| 249 | 1 |  |  |  |  |  | 1 | **CC23** |
| 268 |  | 1 |  |  |  |  | 1 | **CC12** |
| 651 |  |  | 2 |  |  |  | 2 | **/** |
| 680 |  |  | 1 |  |  |  | 1 | **CC17** |
| 938 |  |  |  |  |  | 1 | 1 | **CC1** |
| Total | 18 | 64 | 88 | 1 | 5 | 2 | 178 | **/** |

**Table S1 The distribution of MLST types among various serotypes of GBS.**

MLST, multilocus sequence typing. GBS, group B *Streptococcus*.

**Table S2 Classification results of sporadic STs**

| **Isolate** | **ST** | **CC** | **Subtyping MSP classification^a^(score^b^)** | **Subtyping MSP-M classification^a^(score^b^)** |
| --- | --- | --- | --- | --- |
| 1 | ST2 | CC1 | CC10(1.877) | CC10(2.388) |
| 2 | ST2 | CC1 | CC10(2.129) | CC10(2.513) |
| 3 | ST4 | / | CC10(2.209) | CC10(2.494) |
| 4 | ST24 | CC452 | CC17(2.010) | CC19(2.562) |
| 5 | ST24 | CC452 | CC19(2.160) | CC19(**2.690**) |
| 6 | ST24 | CC452 | CC19(2.165) | CC19(2.566) |
| 7 | ST103 | / | CC12(2.004) | CC12(2.590) |
| 8 | ST156 | CC1 | CC10(2.204) | CC10(2.562) |
| 9 | ST651 | / | CC12(2.120) | CC17(**2.613**) |
| 10 | ST651 | / | CC12(2.141) | CC19(2.486) |
| 11 | ST938 | CC1 | CC19(2.027) | CC23(2.536) |

Abbreviations: CC, clonal complex; ST, sequence type.

Isolates showing logscore values ≥2.6 are shown in bold type.

^a^Only the logscore with the highest value for all spectra originated from the same isolate is shown.

^b^Best score obtained for the subtyping classification of the replicas.

**Table S3 Performance of the pattern recognition models for the differentiation of the main GBS clonal lineages generated by ClinProTools**

| **Method** | **Model** | **Selected peaks** | **Cross validation(%)** | **Recognition value(%)** |
| --- | --- | --- | --- | --- |
| Averaged spectrum | QC | 1 | 69.72 | 81.47 |
|  | SNN | 1 | 49.80 | 38.07 |
|  | GA(10)-KNN1 | 10 | 70.50 | 100.00 |
|  | GA(10)-KNN3 | 9 | 79.73 | 94.52 |
|  | GA(10)-KNN5 | 8 | 79.16 | 95.32 |
|  | GA(10)-KNN7 | 10 | 74.78 | 89.25 |
|  | GA(20)-KNN1 | 20 | 80.89 | 100.00 |
|  | GA(20)-KNN3 | 10 | 73.24 | 94.25 |
|  | GA(20)-KNN5 | 10 | 73.18 | 97.50 |
|  | GA(20)-KNN7 | 11 | 78.53 | 98.18 |
|  | GA(30)-KNN1 | 30 | 67.70 | 100.00 |
|  | GA(30)-KNN3 | 11 | 73.69 | 88.95 |
|  | GA(30)-KNN5 | 14 | 81.82 | 97.50 |
|  | GA(30)-KNN7 | 12 | 76.21 | 94.94 |

Abbreviations: CV, cross-validation; GA,genetic algorithm; QC,Quick Classifier;

RC, recognition capability; SNN, supervised neural network.

a,Maximum number of peaks for model generation;

b,Number of peaks selected by the model.

**Table S4 Wrong classification results of minor STs belonging to five CCs which were not included in the STs of GBS strains in the reference set.**

| **Isolate** |  | **ST** | **CC** | **Subtyping MSP classification^a^(score^b^)** | **Subtyping MSP-M classification^a^(score^b^)** |
| --- | --- | --- | --- | --- | --- |
| 1 |  | ST249 | CC23 | CC19(2.489) | CC17(2.508)  CC12(2.433) |
| 2 |  | ST88 | CC23 | CC12 (2.442) | CC19(2.476) |
| 3 |  | ST268 | CC12 | CC10(2.478) | CC12(2.589)  CC19(2.581) |
| 4 |  | ST8 | CC12 | CC19(2.450)  CC10(2.442) | CC19(2.509)  CC17(2.576) |
| 5 |  | ST188 | CC17 | CC12(2.595) | CC12(2.485)  CC19(2.445) |
| 6 |  | ST680 | CC17 | none | CC12(2.479) |
| 7 |  | ST179 | CC17 | CC23(2.652)  CC12(2.676)  CC19(2.604) | CC12(2.560) |
| 8 |  | ST27 | CC19 | CC12(2.610)  CC10(2.566)  CC23(2.595) | CC12(2.679)  CC12(2.537) |

Abbreviations: CC, clonal complex; ST, sequence type.

^a^Only the logscore with the highest value for all spectra originated from the same isolate is shown.

^b^Best score obtained for the subtyping classification of the replicas.
